# Burnout among medical students in Cyprus: A cross-sectional study

**DOI:** 10.1371/journal.pone.0241335

**Published:** 2020-11-18

**Authors:** Antonios Nteveros, Marios Kyprianou, Artemios Artemiadis, Antrianthi Charalampous, Kallistheni Christoforaki, Stephanos Cheilidis, Orestis Germanos, Panagiotis Bargiotas, Andreas Chatzittofis, Panagiotis Zis

**Affiliations:** Medical School, University of Cyprus, Nicosia, Cyprus; Medical University of Vienna, AUSTRIA

## Abstract

**Objectives:**

The primary aim was to estimate the burnout prevalence among all medical students at the Medical School of the University of Cyprus. Secondary aims were to ascertain the predictors of burnout and its relationship with lifestyle habits, sleep quality and mental health.

**Background:**

Burnout in the healthcare sector has drawn significant scientific attention over the last few years. Recent research underscored the large burden of profession-related burnout among medical students.

**Materials and methods:**

An anonymous questionnaire was administered to all 189 eligible candidates. This included demographic and lifestyle characteristics. Sleep quality was assessed via the Pittsburg Sleep Quality Index, mental health was assessed via the mental health (MH) domain of the 36-item Short Form Health Survey (SF-36) and burnout with the Maslach Burnout Inventory–Student Survey (MBI-SS).

**Results:**

Overall response rate was 96.3%. The burnout prevalence was 18.1%. There was a significant linear effect of between the year of studies and the burnout frequency [F(1) = 5.09, p = 0.024], implying that with increasing academic year there were more students with burnout, especially after the 4th year of education which signifies the beginning of clinical education. Students with burnout were more likely to have poor sleep quality (90.9% vs. 60.8%, odds ratio 4.33, p = 0.023) and worse mental health (MH score 40.2 ± 17.7 vs 62.9 ± 20.3, p<0.001). Alcohol consumers had more symptoms of cynicism and less feelings of efficacy than non-alcohol consumers. Moreover, less feelings of efficacy were significantly associated with more alcohol consumption among alcohol consumers.

**Conclusions:**

Burnout is prevalent in medical students and increases significantly during the clinical years. Students with burnout have worse sleep and mental health and might use alcohol as a coping mechanism. Implementing prevention strategies of burnout may be beneficial.

## Introduction

Burnout is conceptualised as a consequence of inadequately managed chronic workplace stress. It is characterized by a triad of emotional exhaustion, depersonalisation regarding the person’s occupation and a feeling of reduced personal accomplishment. Emotional exhaustion (EE) refers to feelings of being overextended and depleted of emotional resources. Depersonalisation (DP) is characterised by a negative, cynical, and detached response to the people including colleagues and supervisors. A feeling of reduced personal accomplishment (PA) occurs when a person feels less competent regarding professional efficacy [1,2]. Until recently, occupational burnout syndrome was not listed as a legitimate syndrome in the widely accepted classification systems. However, in 2019 burnout was officially included in the ICD-11 as an occupational phenomenon but not as a medical condition [1].

Burnout in the healthcare sector has drawn significant scientific attention over recent years. Most of this research focused on medical professionals or residents. Up to 50% of medical professionals might suffer from burnout [3,4]. However, recent research underscored the large burden of profession-related burnout among medical students [5]. Medical students endure stress due to numerous reasons including the strenuous nature of the medical training program, a requirement to absorb a great deal of information over short periods of time, interaction with death and disease, difficult career choices and financial burdens. These academic, existential and psychological stressors may cause students to experience deteriorating mental health during the course of their studies [6].

Burnout among medical students has many implications due to its association with absenteeism, low morale and dissatisfaction [7]. Burnout may also be associated with adverse outcomes at an individual level including poor decision making, hostility to patients, medical errors, poor relationships with colleagues, depression, anxiety and fatigue, sleep disturbances, alcoholism, drug misuse, and suicidal ideation [8].

The primary aim of this cross-sectional study was to estimate the burnout prevalence among all medical students at the Medical School of the University of Cyprus. Secondary aims included to ascertain the predictors of burnout and its relationship with lifestyle habits, sleep quality and mental health.

## Methods

### Participants

The Medical School Undergraduate Program runs for 6 years (three preclinical and three clinical years). In total 189 students are currently enrolled, all of which were invited to take part in this study.

An anonymous questionnaire was distributed in the form of a hard copy by members of the research team to all eligible candidates during the last week of January 2020 during scheduled classes by the research team members. Students were asked to return the completed questionnaires in a sealed envelope which they placed in a non-transparent empty box, in order to ensure the anonymity of the questionnaire. All candidates were reminded to complete the questionnaire one week after the initial distribution.

Participation in the study was voluntary. All participants provided written informed consent. There was no interference in the study by anyone outside the team of researchers. Therefore, no pressure was applied by clinical or educational supervisors nor anyone else to either take part or decline to participate in the study. The study was approved by the Cyprus National Bioethics Committee (reference number 2019.01.205).

### Assessments

*Demographic characteristics* included age, sex and marital status. *Academic characteristics* included year of studies, academic performance evaluated by the final grade during the previous academic year (applicable to years 2–6) and choice of future medical specialty. *General health characteristics* included smoking status, weekly alcohol unit consumption, weekly physical exercise per week, self-reported weight and height. Body mass index (BMI) was then calculated, and students were categorized as “underweight” (BMI<18.5), “normal” (BMI 18.5 to 25), “overweight” (BMI 25 to 30) or “obese” (BMI>30).

*Mental health (MH)* was evaluated by the MH domain of the 36-item Short Form Health Survey (SF-36) [9]. Students answered the five MH-related items concerning their emotional well-being during the last four weeks in a 6-point Likert-type scale, ranging from 1 (all of the time) to 6 (never). Two of those items (e.g., ‘‘Have you been a happy person?”) were reverse-scored to ensure that a higher item value indicated better MH. Then scores were transformed into a 0 to 100-point scale as follows; 1 = 100, 2 = 80, 3 = 60, 4 = 40, 5 = 20 and 6 = 0. The sum was divided by 5 (averaged). Higher scores denoted better MH. The instrument showed good reliability in this study (Cronbach’s alpha = 0.86).

#### Quality of Sleep (QoS)

The quality of sleep during the last 30 days was measured by the Pittsburg Sleep Quality Index (PSQI) [10]. The PSQI consists of 19 self-reference questions measuring seven domains; subjective sleep quality, sleep latency, sleep duration, habitual sleep efficiency, sleep disturbances, use of sleep medication and daytime dysfunction. Scoring for each component ranges from 0 to 3 and the global score ranges from 0 (indicating high quality of sleep) to 21 (indicating low quality of sleep). A cut-off of 5 or greater is used to identify overall poor sleep quality [11]. The reliability of the instrument in this study was found to be acceptable (Cronbach’s alpha = 0.68).

#### Burnout

The Maslach Burnout Inventory–Student Survey (MBI-SS) was used to evaluate burnout among medical students [7]. This is a 16-item tool, with each item being rated on a 7-point Likert-type scale, ranging from 0 (never) to 6 (everyday). These items produce three subscales: exhaustion (EX), cynicism (CY) and efficacy (EF). As suggested by Schutte et al., one particular CY item (“When I’m in class or I’m studying I don’t want to be bothered”) was removed [12]. The construct and concurrent (i.e. criterion-related) validity and the reliability this instrument were verified (see S1–S5 Tables). The instrument showed good to excellent reliability for this study (Cronbach’s alpha: EX = 0.90, CY = 0.87, EF = 0.80).

Burnout was defined as having EX score higher than the 75^th^ percentile (21 for this study) and in addition to either a CY score higher than the 75^th^ percentile (5 for this study) or EF lower than the 25^th^ percentile (22 for this study), as has been previously suggested [13].

### Statistical analysis

Descriptive statistics of the study sample included absolute and relative (%) frequencies, means, standard deviations, medians, interquartile ranges and ranges. Statistical comparisons were performed between students with and without burnout. Categorical variables were compared using the chi-square test (with Yates correction for 2×2 tables). Continuous variables were not normally distributed and sample sizes of the burnout groups were unequal, therefore they were assessed using the Mann-Whitney U test. Further comparisons for students’ groups by the academic year in the medical school were also performed. Level of significance was set at 0.05. The data were analyzed with SPSS v22.0 for Windows (Armonk, NY: IBM Corp).

## Results

### Description of the study sample

Of the 189 students, 182 participated to the study giving a response rate of 96.3%. Of those who gave a response, 121 (66.5%) were females. Our study population had a mean age of 21.8±3.3. The full characteristics of our study population are summarized in S6 Table. The majority of the students (73.6%) were ascertained to have poor sleep quality.

### Burnout prevalence among medical students

The burnout prevalence among medical students was found to be 18.1% (*having EX score higher than 21 and either CY score higher than 5 or EF lower than 22)*. The burnout prevalences among medical students in each academic year are summarized in Table 1. Ninety three participants (51.1%) had abnormal scores in at least one MBI dimension, 43 (23.6%) in at least two MBI dimensions and 13 (7.1%) in all MBI dimensions.

**Table 1:** Burnout prevalence (n/N, %) among medical students in different academic years (N = 182).

	Total	1^st^	2^nd^	3^rd^	4^th^	5^th^	6^th^	Significance^1^
Students with Burnout	33/182 (18.1)	**1/27 (3.7)**	6/33 (18.2)	3/37 (8.1)	**11/27 (40.7)**	4/29 (13.8)	8/29 (27.6)	p = 0.004
Students without Burnout	149/182 (81.9)	**26/27 (96.3)**	27/33 (81.8)	34/37 (91.9)	**16/27 (59.3)**	25/29 (86.2)	21/29 (72.4)	

^1^Chi-squareexact test.

Bold signify those frequencies lower or higher than the expected based on the adjusted standardized residuals (using the ±1.96 cut-off).

Academic year of study had a significant effect on burnout frequency. Specifically, there were significantly less students with burnout during the 1^st^ academic year and more students with burnout during the 4^th^ year of education (p = 0.004).

Moreover, a significant linear effect was found [F(1) = 5.09, p = 0.024], implying that with increasing academic year there were more students with burnout. This effect was found particularly after the 4^th^ year of education which marks the beginning of clinical study. Looking into the three dimensions separately, EX and CY symptoms also significantly increased with higher academic years, but no significant effects upon EF were found (S7 Table).

### Burnout associations

Table 2 presents the univariate associations of burnout among medical students. There were more students with burnout during the clinical years (4th to 6th) than during the non-clinical years of education (1st to 3rd) [27.1% vs. 10.3%, OR 3.23, 95%CI: 1.44–7.26, F(1) = 7.471, p = 0.006*]. Moreover, students with burnout had worse mental health (U = 1002, z = 5.33, p<0.001) and were more likely to have poor sleep quality than students with no burnout [90.9% vs. 60.8%, OR 4.33, 95%CI: 1.26–14.9, F(1) = 5.161, p = 0.023*]. No significant effect of sex, age, marital status, academic performance, decision on specialty, exercise, smoking, alcohol or BMI were found in relation to burnout (S8 and S9 Tables).

**Table 2:** Burnout significant associations in medical students.

	Students with Burnout (N = 33)	Students without Burnout (N = 149)	Significance Tests^1^
Academic Period			F(1) = 7.471, p = 0.006*
*Non-Clinical (1*^*st*^*-3*^*rd*^ *year)*	10/33 (30.3)	87/149 (58.4)	
*Clinical (4*^*th*^*-6*^*th*^ *year)*	23/33 (69.7)	62/149 (41.6)	
Mental Health score	40.2 ± 17.7	62.9 ± 20.3	U = 1002, z = 5.33, p<0.001*
Sleep Quality score	8.6 ± 3.5	6.1 ± 2.9	U = 1452.5, z = 3.67 p<0.001*
Poor Sleep Quality	30/33 (90.9)	104/149 (69.8)	F(1) = 5.161, p = 0.023*

^1^Chi-square tests (Yates correction for 2×2 tables) for categorical data, Mann-Whitney U test for numerical data.

*p≤0.05.

Alcohol consumers, however, had more symptoms of CY and less feelings of EF than non-alcohol consumers (S10 Table). Moreover, reduced feelings of EF were significantly associated with higher levels of alcohol consumption among those who alcohol (S11 Table).

## Discussion

This study investigated the burnout prevalence among medical students at the University of Cyprus and explored its predictors.

We found that almost one in five study participants is “burned-out”. In the literature, burnout prevalence rates of up to 75.2% have been reported in the medical students [14,15]. However, there is an enormous level of heterogeneity in international reporting of prevalence rates of burnout in medical students because the tools used to measure burnout differ and also because the exact definition utilised differs even when using the same instrument. In addition, discrepancies with other studies may be accounted for by differences in curricula, means of medical education, available facilities, cross-cultural characteristics and other factors.

We found that year of medical education was the only significant predictor of burnout, which increased towards the end of studies. This is in keeping with most cross-sectional studies [6,16–22]. It is possible that this effect is related to the impact of exposure to real clinical settings and interaction with patients during these years. In contrast to these findings, Dos Santos Boni et al. showed that the prevalence of burnout was higher in the first year of medical school than the other years [23], a finding that was attributed to high levels of stress prior to the entry exams [23].

In our study population, demographics were not found to have any statistically significant effect on the prevalence of burnout. This is in contrast with other studies, which found that females are affected by EX to a greater extent than men [15,24–28].

Even though alcohol consumption was not significantly associated with burnout in our study, alcohol consumers had higher scores in the CY and lower scores in the EF dimensions of the MBI-SS compared to non-alcohol consumers. Also, less feelings of EF were significantly associated with more alcohol consumption amongst those who consume alcohol. A possible explanation for this is that alcohol consumption might act as a coping strategy, as is indicated by other studies [29–31]. Physical exercise is another coping strategy, which has been reported to reduce the probability of burnout [29,32]. However, in our study population this was not confirmed.

Burnout has a significant impact in students’ behaviors. Our finding that students with burnout have overall worse mental health is in keeping with what has been previously been reported. Fitzpatrick et al. showed that burnout is a significant risk factor for the development of depression in medical students and that higher levels of burnout are associated with less help-seeking behavior for their mental health problems [17]. Furthermore, it has been shown that medical students with higher burnout risk have decreased engagement with studying and are more likely to engage in unprofessional behaviors [33,34].

In this study the quality of sleep during the last 30 days was measured via the PSQI. The majority of our study population (73.6%) had poor sleep quality. As was shown in other studies [31,35] students with burnout were more likely to have poor sleep quality compared to non-burned out students. A possible explanation is that poor sleep quality reflects the mental distress of students with burnout [36].

### Limitations

Our study findings should be interpreted with caution due to some limitations. Firstly, the cross-sectional nature of this study does not permit etiological inferences. Secondly, we used self-reporting questionnaires to assess MH and sleep which asked questions relating to a period of weeks prior to participation. Such questionnaires carry a risk of recall bias, which should be acknowledged. Additionally, the questionnaires captured a period of four weeks before the last week of January which means that one week of the holidays was included. The fact that different sleep patterns are seen during a holiday and a non-holiday period might account for the questionable reliability of the PSQI instrument. On the other hand, burnout and MH are more complicated characteristics resulting from a constellation of environmental and personal factors, thus they would be less affected by the holiday effect described above. Longitudinal data would further validate our findings by eliminating the confounding factors of period of assessments, personality characteristics and other residual confounding in general. Finally, although our response rate was excellent the absolute sample size is relatively small.

### Directions for the future

Studies in qualified doctors have shown that burnout ranges from 18 to 82% [37,38] which has a significant effect on overall mental health and work engagement [39,40]. Furthermore, burnout may lead to medical negligence and malpractice litigation, as well as suboptimal patient care practices and attitudes [38,41]. Residency programme directors are likely to inherit medical school graduates with a substantial burden of burnout symptoms [42]. Therefore, prevention strategies of burnout are needed at all levels, from medical students to specialists. A consistent finding across burnout studies in medical students is that it occurs during the clinical years. Program directors at medical schools should offer psychological support and services to students with burnout the medical education years.

### Conclusion

In conclusion, burnout is prevalent among medical students especially during the clinical years of education. Furthermore, we showed that burnout affects sleep and overall mental health as well as it is linked to maladaptive coping behaviors such as increased alcohol consumption. Prevention strategies of burnout are needed during medical studies.

## Supporting information

S1 Table Rotated factor loadings of the principal component analysis (PCA) for the 15 items of the Maslach Burnout Inventory–Student Survey (MBI-SS). (DOCX)

S2 Table Descriptive measures of the MBI-SS subscale scores. (DOCX)

S3 Table Cronbach’s alphas if items deleted for each MBI-SS subscale. (DOCX)

S4 Table Correlations between MBI-SS subscales (Pearson’s rho). (DOCX)

S5 Table Correlations between MBI-SS subscales and academic performance, mental health and sleep quality (Pearson’s rho). (DOCX)

S6 Table Study population’s characteristics. (DOCX)

S7 Table MBI-SS subscale scores among academic years in the medical school. (DOCX)

S8 Table Non-significant burn-out associations in medical students. (DOCX)

S9 Table Gender differences for MBI-SS subscale scores. (DOCX)

S10 Table MBI-SS subscale scores and smoking, alcohol consumption and regular exercise status. (DOCX)

S11 Table Correlations between MBI-SS subscales and alcohol consumption units, total exercise per week in minutes and BMI (Pearson’s rho). (DOCX)

S1 Data (SAV)
