# Supplementary material for: Burnout among medical students in Cyprus: A cross-sectional study
Source: PLoS One. 2020 Nov 18;15(11):e0241335. doi: 10.1371/journal.pone.0241335 (PMC7673498; doi:10.1371/journal.pone.0241335)
Supplement: S1 Table — (DOCX) [file pone.0241335.s001.docx]

**Table S1.** Rotated factor loadings of the principal component analysis (PCA) for the 15 items of the Maslach Burnout Inventory – Student Survey (MBI-SS)

| Items | Exhaustion | Cynicism | Efficacy |
| --- | --- | --- | --- |
| MBI-SS 1 | 0.866 |  |  |
| MBI-SS 2 | 0.758 |  |  |
| MBI-SS 3 | 0.838 |  |  |
| MBI-SS 4 | 0.732 |  |  |
| MBI-SS 5 | 0.865 |  |  |
| MBI-SS 6 |  | 0.826 |  |
| MBI-SS 7 |  | 0.825 |  |
| MBI-SS 8 |  | 0.808 |  |
| MBI-SS 9 |  | 0.714 |  |
| MBI-SS 10 |  |  | 0.677 |
| MBI-SS 11 |  |  | 0.644 |
| MBI-SS 12 |  |  | 0.783 |
| MBI-SS 13 |  |  | 0.717 |
| MBI-SS 14 |  |  | 0.501 |
| MBI-SS 15 |  |  | 0.813 |
| Eigenvalues | 3.713 | 2.952 | 3.06 |
| % Variance | 24.8 | 19.7 | 20.4 |
| Cronbach’s alpha | 0.902 | 0.866 | 0.796 |
| Bartlett’s test: F(105) = 1466.4, p<0.001  Determinant: 0  Kaiser-Meyer-Olkin: 0.829  Communalities average: 0.65 | | | |
