# Supplementary material for: Burnout among medical students in Cyprus: A cross-sectional study
Source: PLoS One. 2020 Nov 18;15(11):e0241335. doi: 10.1371/journal.pone.0241335 (PMC7673498; doi:10.1371/journal.pone.0241335)
Supplement: S2 Table — (DOCX) [file pone.0241335.s002.docx]

**Table S2.** Descriptive measures of the MBI-SS subscale scores

|  | **Mean (SD)** | **Median (IQR)** | **Range** | **Theoretical Range** |
| --- | --- | --- | --- | --- |
| Exhaustion | 15 (7.7) | 15 (12) | 0-29 | 0-30 |
| Cynicism | 3.3 (4.8) | 1 (4.25) | 0-21 | 0-24 |
| Efficacy | 25.8 (6.2) | 27 (8) | 1-36 | 0-36 |
