# Supplementary material for: Burnout among medical students in Cyprus: A cross-sectional study
Source: PLoS One. 2020 Nov 18;15(11):e0241335. doi: 10.1371/journal.pone.0241335 (PMC7673498; doi:10.1371/journal.pone.0241335)
Supplement: S3 Table — (DOCX) [file pone.0241335.s003.docx]

**Table S3.** Cronbach’s alphas if items deleted for each MBI-SS subscale

| Exhaustion | Cronbach’s alpha | Cynicism | Cronbach’s alpha | Efficacy | Cronbach’s alpha |
| --- | --- | --- | --- | --- | --- |
| MBI-SS 1 | 0.877 | MBI-SS 6 | 0.815 | MBI-SS 10 | 0.765 |
| MBI-SS 2 | 0.885 | MBI-SS 7 | 0.796 | MBI-SS 11 | 0.777 |
| MBI-SS 3 | 0.884 | MBI-SS 8 | 0.837 | MBI-SS 12 | 0.753 |
| MBI-SS 4 | 0.892 | MBI-SS 9 | 0.857 | MBI-SS 13 | 0.757 |
| MBI-SS 5 | 0.864 |  |  | MBI-SS 14 | 0.800 |
|  |  |  |  | MBI-SS 15 | 0.730 |
