# Supplementary material for: Burnout among medical students in Cyprus: A cross-sectional study
Source: PLoS One. 2020 Nov 18;15(11):e0241335. doi: 10.1371/journal.pone.0241335 (PMC7673498; doi:10.1371/journal.pone.0241335)
Supplement: S4 Table — (DOCX) [file pone.0241335.s004.docx]

**Table S4.** Correlations between MBI-SS subscales (Pearson’s rho)

| Items | Exhaustion | Cynicism | Efficacy |
| --- | --- | --- | --- |
| Exhaustion | 1 |  |  |
| Cynicism | 0.476* | 1 |  |
| Efficacy | -0.206* | -0.31* | 1 |

*p≤0.05
