# Supplementary material for: Burnout among medical students in Cyprus: A cross-sectional study
Source: PLoS One. 2020 Nov 18;15(11):e0241335. doi: 10.1371/journal.pone.0241335 (PMC7673498; doi:10.1371/journal.pone.0241335)
Supplement: S5 Table — (DOCX) [file pone.0241335.s005.docx]

**Table S5.** Correlations between MBI-SS subscales and academic performance, mental health and sleep quality (Pearson’s rho)

| Items | Academic Performance | Mental Health | Sleep Quality Problems |
| --- | --- | --- | --- |
| Exhaustion | 0.237* | -0.581* | 0.425* |
| Cynicism | -0.12 | -0.472* | 0.307* |
| Efficacy | 0.129 | 0.398* | -0.297* |

*p≤0.05
