# Supplementary material for: Burnout among medical students in Cyprus: A cross-sectional study
Source: PLoS One. 2020 Nov 18;15(11):e0241335. doi: 10.1371/journal.pone.0241335 (PMC7673498; doi:10.1371/journal.pone.0241335)
Supplement: S6 Table — (DOCX) [file pone.0241335.s006.docx]

**Table S6.** Study population’s characteristics.

|  | n/N (%) | Mean (SD) | Median (IQR) | Range |
| --- | --- | --- | --- | --- |
| Sex  *Males*  *Females* | 61/182 (33.5)  121/182 (66.5) | - | - | - |
| Age (years) | - | 21.8 (3.3) | 21 (3) | 18-52 |
| Marital Status  *Unmarried*  *Married* | 176/180 (97.8)  4/180 (2.2) | - | - | - |
| Year of Studies  *First*  *Second*  *Third*  *Fourth*  *Fifth*  *Sixth* | 27/182 (14.8)  33/182 (18.1)  37/182 (20.3)  27/182 (14.8)  29/182 (15.9)  29/182 (15.9) | - | - | - |
| Previous year’s grade^1^ | - | 8 (0.8) | 8 (1) | 5 – 9.5 |
| Decided on specialty | 38/182 (20.9) | - | - | - |
| Regular exercise (yes) | 98/182 (53.8) | - | - | - |
| Total exercise (per week)^2^ | - | 3hr 48min (2hrs 24min) | 3 (3) | 42min-12hrs |
| Smoking^3^  *No*  *Yes*  *Ex* | 143/177 (80.8)  28/177 (15.8)  6/177 (3.4) | - | - | - |
| Alcohol consumption^4^  *No*  *Yes* | 101/181 (55.8)  80/181 (44.2) | - | - | - |
| Alcohol consumption (IU)^5^ | - | 4.9 (5.1) | 3 (3) | 0.3-30 |
| Weight (Kg)^4^ | - | 66 (15.3) | 62 (20) | 42-120 |
| BMI (Kg/m2)^4^ | - | 23 (3.8) | 22.2 (4.8) | 16.2-36 |
| BMI Categories^4^  *Underweight (<18.5 Kg/m2)*  *Normal (18.5-24.9 kg/m2)*  *Overweight (25-29.9 kg/m2)*  *Obese (>30 kg/m2)* | 12/181 (6.6)  125/181 (69.1)  34/181 (18.8)  10/181 (5.5) | - | - | - |
| Exhaustion | - | 15 (7.7) | 15 (12) | 0-29 |
| Cynicism | - | 3.3 (4.8) | 1 (4.25) | 0-21 |
| Efficacy | - | 25.8 (6.2) | 27 (8) | 1-36 |
| Mental Health score | - | 58.8 (21.6) | 60 (33) | 0-100 |
| Sleep Quality score | - | 6.6 (3.1) | 6 (5) | 0-16 |
| Poor Sleep Quality | 134/182 (73.6) | - | - | - |

^1^Missing values: 27 missing due to the exclusion of the 1^st^ year students and 6 (3.3%) students do not answered

^2^Calculated for the 101 students who reported engagement in regular weekly exercise

^3^Missing values: 5 (2.7%)

^4^Missing values: 1 (0.5%)

^5^Calculated for the 80 students who answered “yes” for alcohol consumption

IQR; Interquartile range, SD; Standard Deviation
