# Supplementary material for: Burnout among medical students in Cyprus: A cross-sectional study
Source: PLoS One. 2020 Nov 18;15(11):e0241335. doi: 10.1371/journal.pone.0241335 (PMC7673498; doi:10.1371/journal.pone.0241335)
Supplement: S7 Table — (DOCX) [file pone.0241335.s007.docx]

**Table S7.** MBI-SS subscale scores among academic years in the medical school

|  | 1^st^ (N=27) | 2^nd^  (N=33) | 3^rd^  (N=37) | 4^th^  (N=27) | 5^th^  (N=29) | 6^th^  (N=29) | Significance Tests^1^ |
| --- | --- | --- | --- | --- | --- | --- | --- |
| Exhaustion | 10.4 (6.6) | 13.4 (7.4) | 15.1 (7.3) | 19.6 (7.7) | 16.1 (6.7) | 15.5  (8) | **F(5,176)=4.703, p<0.001*** |
| Cynicism | 1.9 (3.8) | 2.5 (4.6) | 2.4 (3.6) | 3.7 (5.4) | 3.6  (5) | 5.7 (5.7) | **F(5,151)=2.534, p=0.031*^2^** |
| Efficacy | 26.6 (4.5) | 24.1 (7.1) | 26.5 (5.3) | 25.4 (5.8) | 28  (5.8) | 24.1  (7.6) | F(5,176)=1.945, p=0.089 |

Values represent means (standard deviations)

^1^ANOVA test

^2^Brown-Forsythe test was applied because the Levene test of homogeneity was significant (p=0.042)
