# Supplementary material for: Burnout among medical students in Cyprus: A cross-sectional study
Source: PLoS One. 2020 Nov 18;15(11):e0241335. doi: 10.1371/journal.pone.0241335 (PMC7673498; doi:10.1371/journal.pone.0241335)
Supplement: S8 Table — (DOCX) [file pone.0241335.s008.docx]

**Table S8.** Non-significant burn-out associations in medical students

|  | Students with Burnout (N=33) | Students without Burnout (N=149) | Significance Tests^1^ |
| --- | --- | --- | --- |
| Females | 24/33 (72.7) | 97/149 (65.1) | F(1)=0.404, p=0.525 |
| Age (years) | 22.1 ± 1.6 | 21.8 ± 3.6 | U=1945.5, z=1.89  p=0.059 |
| Marital Status  *Unmarried*  *Married with Children*  *Unmarried with children* | 33/33 (100)  0/33 (0)  0/33 (0) | 141/147 (95.9)  4/147 (2.7)  2/147 (1.4) | F(2)=1.393, p=0.498 |
| Final Grade (previous year) | 8.1 ± 0.9 | 7.9 ± 0.8 | U=1688.5, z=086  p=0.391 |
| Decided on specialty | 3/33 (9.1) | 35/149 (23.5) | F(1)=2.575, p=0.109 |
| Regular exercise (yes) | 16/33 (48.5) | 82/149 (55) | F(1)=0.24, p=0.624 |
| Total exercise (per week) | 3hrs 48mins ± 2hrs 24mins | 8hrs 48mins ± 2hrs 25mins | U=649.5, z=0.06  p=0.95 |
| Currently Smoking  *No*  *Yes* | 27/32 (78.1)  5/32 (15.6) | 122/145 (81.4)  23/145 (15.9) | F(1)=0, p=1.0 |
| Alcohol consumption  *No*  *Yes* | 14/32 (43.8)  18/32 (56.3) | 87/149 (58.4)  62/149 (41.6) | F(1)=1.734, p=0.188 |
| Alcohol consumption in IU | 4.6 ± 4.3 | 5 ± 5.4 | U=513, z=0.52  p=0.6 |
| Weight (Kg) | 67.5 ± 17.2 | 65.6 ± 14.9 | U=2307.5, z=0.5  p=0.621 |
| BMI (Kg/m2) | 23.4 ± 4 | 22.9 ± 3.8 | U=2304.5, z=0.51  p=0.613 |
| BMI Categories  *Underweight (<18.5 Kg/m2)*  *Normal (18.5-24.9 kg/m2)*  *Overweight (25-29.9 kg/m2)*  *Obese (>30 kg/m2)* | 1/33 (3)  22/33 (66.7)  7/33 (21.2)  3/33 (9.1) | 11/148 (7.4)  103/148 (69.6)  27/148 (18.2)  7/148 (4.7) | F(3)=1.878, p=0.598 |
