# Supplementary material for: Burnout among medical students in Cyprus: A cross-sectional study
Source: PLoS One. 2020 Nov 18;15(11):e0241335. doi: 10.1371/journal.pone.0241335 (PMC7673498; doi:10.1371/journal.pone.0241335)
Supplement: S9 Table — (DOCX) [file pone.0241335.s009.docx]

**Table S9**. Gender differences for MBI-SS subscale scores

|  | Males (N=61) | Females (N=121) | Significance Tests^1^ |
| --- | --- | --- | --- |
| Exhaustion | 14.9 (7.6) | 15 (7.7) | 0.967 |
| Cynicism | 4 (5) | 2.9 (4.6) | 0.091 |
| Efficacy | 25 (5.9) | 26.2 (6.3) | 0.103 |

Values represent means (standard deviations)

^1^Mann-Whitney U tests
