# Supplementary material for: Burnout among medical students in Cyprus: A cross-sectional study
Source: PLoS One. 2020 Nov 18;15(11):e0241335. doi: 10.1371/journal.pone.0241335 (PMC7673498; doi:10.1371/journal.pone.0241335)
Supplement: S10 Table — (DOCX) [file pone.0241335.s010.docx]

**Table S10**. MBI-SS subscale scores and smoking, alcohol consumption and regular exercise status

|  | Smokers (N=34) | No or Past Smokers (N=143) | Significance Tests^1^ |
| --- | --- | --- | --- |
| Exhaustion | 15.9 (7) | 15 (7.7) | 0.404 |
| Cynicism | 4 (5.4) | 3.1 (4.6) | 0.391 |
| Efficacy | 24.4 (7.5) | 26 (5.8) | 0.307 |
|  | Alcohol Consumers (N=80) | No Alcohol Consumers (N=101) | Significance Tests^1^ |
| Exhaustion | 15.7 (7.3) | 14.3 (7.8) | 0.198 |
| Cynicism | 4.4 (5.2) | 2.2 (3.9) | **0.002*** |
| Efficacy | 24.2 (6.7) | 27 (5.5) | **0.004*** |
|  | Regular Exercise  (N=101) | No Regular Exercise (N=81) | Significance Tests^1^ |
| Exhaustion | 14.5 (7.5) | 15.6 (7.8) | 0.352 |
| Cynicism | 3.5 (5) | 3 (4.5) | 0.8 |
| Efficacy | 26.3 (5.4) | 25.2 (7) | 0.462 |

Values represent means (standard deviations)

^1^Mann-Whitney U tests

*p≤0.05
