# Supplementary material for: Burnout among medical students in Cyprus: A cross-sectional study
Source: PLoS One. 2020 Nov 18;15(11):e0241335. doi: 10.1371/journal.pone.0241335 (PMC7673498; doi:10.1371/journal.pone.0241335)
Supplement: S11 Table — (DOCX) [file pone.0241335.s011.docx]

**Table S11.** Correlations between MBI-SS subscales and alcohol consumption units, total exercise per week in minutes and BMI (Pearson’s rho)

| Items | Alcohol Consumption | Exercise Time | BMI |
| --- | --- | --- | --- |
| Exhaustion | -0.141 | 0.039 | 0.064 |
| Cynicism | 0.051 | 0.026 | 0.124 |
| Efficacy | **-0.347*** | -0.044 | -0.145 |

*p≤0.05
